# Supplementary material for: Landscape structure shapes the diversity of tree seedlings at multiple spatial scales in a fragmented tropical rainforest
Source: PLoS One. 2021 Jul 16;16(7):e0253284. doi: 10.1371/journal.pone.0253284 (PMC8284835; doi:10.1371/journal.pone.0253284)

**Supporting file**

**Landscape structure shapes the diversity of tree seedlings at multiple spatial scales in a fragmented tropical rainforest**

Nicasio-Arzeta^1,2^, S., I. Zermeño-Hernández^3^, S. Maza-Villalobos^4^ and J. Benítez-Malvido^2^

^1^Programa de Doctorado en Ciencias Biomédicas, Universidad Nacional Autónoma de México (UNAM), Ciudad de México, México

^2^Instituto de Investigaciones en Ecosistemas y Sustentabilidad, Universidad Nacional Autónoma de México. Antigua Carretera a Pátzcuaro no. 8701. Ex-Hacienda de San José de la Huerta, 58190, Morelia, Michoacán, México.

^3^Instituto de Investigaciones sobre los Recursos Naturales (INIRENA), Universidad Michoacana de San Nicolás de Hidalgo. Avenida San Juanito Itzícuaro SN, 58330 Morelia Michoacán, Mexico.

^4^CONACyT-ECOSUR. Carretera Antiguo Aeropuerto Km. 2.5, Centro, 30700, Tapachula, Chiapas, México.

Table A. Coefficient of determination (R^2^) and p value (in parenthesis) between α- and β-diversity metrics and patch size and shape.

| Diversity | Diversity order | Patch size | Patch shape |
| --- | --- | --- | --- |
| α | ***All (^0^α)*** | 0.071 (0.16) | 0.054 (0.195) |
|  | ***Typical (^1^α)*** | 0.103 (0.122) | 0.037 (0.229) |
|  | ***Dominant (^2^α)*** | 0.098 (0.128) | 0.001 (0.33) |
| β | ***All (^0^β)*** | -0.007 (0.358) | 0.023 (0.264) |
|  | ***Typical (^1^β)*** | 0.043 (0.217) | 0.047 (0.208) |
|  | ***Dominant (^2^β)*** | -0.027 (0.45) | -0.01 (0.373) |

Table B. Abbreviation and description of the landscape metrics employed in the analysis

| Landscape metric | Abbreviation | Formula | Description |
| --- | --- | --- | --- |
| Aggregation index | AI | $AI=100\times\left[ \frac{g_{ii}}{max\to g_{ii}} \right]$ | Where *g_ii_* is the number of joins between pixels of forest patch *i* and *max→g_ii_* is the maximum number of joins between pixels of forest patch *i* based on the single-count method |
| Edge contrast index | EC | $EC=100\times\left[ \frac{\sum_{k=1}^{m} p_{ijk}\cdot d_{ik}}{p_{ij}} \right]$ | Where *p_ijk_* is the edge length (m) of patch *ij* adjacent to cover type *k*, *d_ik_* is the edge contrast weight between patch *i* and cover *k*, and *p_ij_* is the length of perimeter of patch *ij* |
| Euclidean-nearest neighbor distance | ENN | $ENN=\frac{\sum_{i=1}^{n} h_{ij}}{n_{h}}$ | Where *h_ij_* is the edge-to-edge distance between two patches *j* of the land cover *i*, and *n_h_* is the number of distances recorded |
| Forest cover | FC | $FC=100\times\left[ \frac{\sum_{j=1}^{n} a_{i}}{A} \right]$ | Where *a* is the area (m^2^) of forest patch *i* and *A* the is the total landscape area |
| Secondary vegetation | SV | $SV=100\times\left[ \frac{\sum_{j=1}^{n} a_{i}}{A} \right]$ | Where *a* is the area (m^2^) of cover patch *i* and *A* the is the total landscape area |
| Patch density | PD | $PD=100\times\left[ \frac{n_{i}}{A}\times\left( 10,000 \right) \right]$ | Where *n_i_* is the total number of patches in the landscape of the land cover *i* and *A* is the total landscape area (m^2^) |

Table C. Correlation values among landscape metrics across the 13 spatial scales employed in the multi-scale analysis in a fragmented tropical forest in southeastern Mexico. Strong correlations (-0.7 > r > 0.7) are in bold.

| Landscape radius |  | *AI* | *EC* | *PD* | *FC* |
| --- | --- | --- | --- | --- | --- |
| 300m | *EC* | -0.475 |  |  |  |
|  | *PD* | -0.582 | 0.283 |  |  |
|  | *FC* | 0.497 | -0.621 | -0.303 |  |
|  | *SF* | 0.333 | -0.102 | -0.464 | 0.202 |
| 400m | *EC* | -0.071 |  |  |  |
|  | *PD* | -0.618 | -0.373 |  |  |
|  | *FC* | 0.573 | -0.477 | -0.145 |  |
|  | *SF* | -0.066 | -0.278 | 0.186 | 0.127 |
| 500m | *EC* | 0.171 |  |  |  |
|  | *PD* | -0.57 | -0.568 |  |  |
|  | *FC* | 0.303 | -0.44 | 0.256 |  |
|  | *SF* | -0.188 | -0.156 | -0.119 | -0.273 |
| 600m | *EC* | 0.084 |  |  |  |
|  | *PD* | -0.135 | -0.334 |  |  |
|  | *FC* | 0.155 | -0.459 | 0.413 |  |
|  | *SF* | -0.413 | -0.123 | 0.076 | -0.417 |
| 700m | *EC* | -0.051 |  |  |  |
|  | *PD* | -0.088 | -0.339 |  |  |
|  | *FC* | 0.079 | -0.474 | 0.503 |  |
|  | *SF* | -0.528 | -0.073 | -0.189 | -0.438 |
| 800m | *EC* | -0.102 |  |  |  |
|  | *PD* | -0.299 | -0.382 |  |  |
|  | *FC* | -0.062 | -0.492 | 0.67 |  |
|  | *SF* | -0.307 | 0.033 | -0.498 | -0.398 |
| 900m | *EC* | -0.331 |  |  |  |
|  | *PD* | -0.031 | -0.225 |  |  |
|  | *FC* | **0.875** | -0.33 | 0.148 |  |
|  | *SF* | -0.38 | 0.056 | -0.237 | -0.451 |
| 1000m | *EC* | -0.32 |  |  |  |
|  | *PD* | -0.355 | -0.226 |  |  |
|  | *FC* | **0.882** | -0.354 | -0.124 |  |
|  | *SF* | -0.282 | 0.068 | -0.145 | -0.426 |
| 1100m | *EC* | -0.195 |  |  |  |
|  | *PD* | -0.142 | -0.373 |  |  |
|  | *FC* | **0.838** | -0.264 | 0.293 |  |
|  | *SF* | -0.265 | 0.128 | -0.211 | -0.371 |
| 1200m | *EC* | -0.16 |  |  |  |
|  | *PD* | -0.039 | -0.687 |  |  |
|  | *FC* | **0.817** | -0.376 | 0.291 |  |
|  | *SF* | -0.244 | 0.27 | -0.48 | -0.36 |
| 1300m | *EC* | -0.321 |  |  |  |
|  | *PD* | -0.014 | -0.551 |  |  |
|  | *FC* | **0.859** | -0.498 | 0.207 |  |
|  | *SF* | -0.291 | 0.444 | -0.291 | -0.427 |
| 1400m | *EC* | -0.361 |  |  |  |
|  | *PD* | -0.02 | -0.609 |  |  |
|  | *FC* | **0.906** | -0.525 | 0.019 |  |
|  | *SF* | -0.439 | 0.526 | -0.233 | -0.507 |
| 1500m | *EC* | -0.34 |  |  |  |
|  | *PD* | 0.193 | -0.597 |  |  |
|  | *FC* | **0.932** | -0.511 | 0.209 |  |
|  | *SF* | -0.539 | 0.583 | -0.218 | -0.56 |

Table D. Percentage of landscape overlap among circular buffers of different radius (300 to1500-m radius, at 100 m intervals) in a fragmented tropical forest in southeastern Mexico. We divided the total overlapped area by the sum of the 16 buffers’ area for each landscape radius.

| Landscape radius | Overlapping (%) |
| --- | --- |
| 300m | 2.139 |
| 400m | 7.56 |
| 500m | 13.159 |
| 600m | 19.078 |
| 700m | 25.026 |
| 800m | 31.037 |
| 900m | 37.676 |
| 1000m | 44.865 |
| 1100m | 52.491 |
| 1200m | 60.964 |
| 1300m | 70.349 |
| 1400m | 80.433 |
| 1500m | 91.091 |

Table E. Moran’s I autocorrelation tests between the distance of sampling sites of tree seedling community and the reported α- and β-diversity (*^0^D* = all species, *^1^D* = typical species and *^2^D* = abundant species) in a fragmented tropical forest in southeastern Mexico.

| Diversity | Order | Moran’s I ± standard deviation | P value |
| --- | --- | --- | --- |
| α | ***^0^α*** | -0.043 **±** 0.065 | 0.721 |
|  | ***^1^α*** | -0.055 **±** 0.065 | 0.853 |
|  | ***^2^α*** | -0.063 **±** 0.065 | 0.957 |
| β | ***^0^β*** | -0.077 **±** 0.059 | 0.861 |
|  | ***^1^β*** | -0.161 **±** 0.065 | 0.147 |
|  | ***^2^β*** | -0.175 **±** 0.063 | 0.088 |

Table F. Moran’s I autocorrelation tests between the distance of sampling sites of tree seedling community and landscape metrics across the 13 spatial scales employed in the multi-scale analysis in a fragmented tropical forest in southeastern Mexico.

| Landscape metric | Landscape radius | Moran ± standard deviation | *P* value |
| --- | --- | --- | --- |
| AI | 300m | -0.025 ± 0.065 | 0.523 |
|  | 400m | -0.021 ± 0.065 | 0.485 |
|  | 500m | -0.033 ± 0.062 | 0.59 |
|  | 600m | 0.058 ± 0.064 | 0.054 |
|  | 700m | 0.053 ± 0.066 | 0.068 |
|  | 800m | -0.092 ± 0.066 | 0.698 |
|  | 900m | -0.004 ± 0.062 | 0.31 |
|  | 1000m | -0.002 ± 0.062 | 0.293 |
|  | 1100m | -0.044 ± 0.061 | 0.71 |
|  | 1200m | -0.036 ± 0.06 | 0.607 |
|  | 1300m | -0.047 ± 0.063 | 0.75 |
|  | 1400m | -0.047 ± 0.064 | 0.762 |
|  | 1500m | -0.066 ± 0.063 | 0.99 |
| EC | 300m | -0.035 ± 0.064 | 0.624 |
|  | 400m | -0.044 ± 0.064 | 0.722 |
|  | 500m | -0.062 ± 0.065 | 0.938 |
|  | 600m | -0.083 ± 0.065 | 0.806 |
|  | 700m | -0.111 ± 0.065 | 0.493 |
|  | 800m | -0.125 ± 0.065 | 0.373 |
|  | 900m | -0.111 ± 0.065 | 0.5 |
|  | 1000m | -0.071 ± 0.066 | 0.951 |
|  | 1100m | -0.01 ± 0.066 | 0.385 |
|  | 1200m | 0.044 ± 0.066 | 0.093 |
|  | 1300m | 0.051 ± 0.067 | 0.077 |
|  | 1400m | 0.057 ± 0.067 | 0.063 |
|  | 1500m | 0.034 ± 0.066 | 0.131 |
| PD | 300m | -0.024 ± 0.056 | 0.446 |
|  | 400m | -0.035 ± 0.066 | 0.635 |
|  | 500m | -0.076 ± 0.064 | 0.887 |
|  | 600m | -0.114 ± 0.065 | 0.469 |
|  | 700m | -0.028 ± 0.063 | 0.541 |
|  | 800m | -0.15 ± 0.061 | 0.176 |
|  | 900m | -0.017 ± 0.063 | 0.432 |
|  | 1000m | -0.023 ± 0.065 | 0.507 |
|  | 1100m | 0.04 ± 0.063 | 0.091 |
|  | 1200m | 0.038 ± 0.065 | 0.109 |
|  | 1300m | -0.124 ± 0.066 | 0.386 |
|  | 1400m | -0.038 ± 0.059 | 0.629 |
|  | 1500m | -0.038 ± 0.061 | 0.638 |
| FC | 300m | **0.067 ± 0.067** | **0.044*** |
|  | 400m | 0.04 ± 0.064 | 0.097 |
|  | 500m | -0.018 ± 0.058 | 0.4 |
|  | 600m | -0.043 ± 0.048 | 0.625 |
|  | 700m | -0.051 ± 0.04 | 0.7 |
|  | 800m | -0.052 ± 0.032 | 0.639 |
|  | 900m | -0.024 ± 0.045 | 0.346 |
|  | 1000m | 0.005 ± 0.06 | 0.228 |
|  | 1100m | 0.009 ± 0.065 | 0.246 |
|  | 1200m | -0.001 ± 0.065 | 0.319 |
|  | 1300m | -0.012 ± 0.065 | 0.402 |
|  | 1400m | -0.029 ± 0.064 | 0.557 |
|  | 1500m | -0.05 ± 0.063 | 0.793 |
| SF | 300m | -0.018 ± 0.066 | 0.459 |
|  | 400m | -0.044 ± 0.066 | 0.73 |
|  | 500m | -0.038 ± 0.065 | 0.657 |
|  | 600m | -0.038 ± 0.064 | 0.652 |
|  | 700m | -0.069 ± 0.06 | 0.971 |
|  | 800m | -0.075 ± 0.06 | 0.886 |
|  | 900m | -0.071 ± 0.059 | 0.946 |
|  | 1000m | -0.071 ± 0.058 | 0.935 |
|  | 1100m | -0.073 ± 0.058 | 0.911 |
|  | 1200m | -0.073 ± 0.059 | 0.911 |
|  | 1300m | -0.074 ± 0.06 | 0.903 |
|  | 1400m | -0.078 ± 0.061 | 0.855 |
|  | 1500m | -0.069 ± 0.062 | 0.972 |

* Significant autocorrelation values

Table G. Moran’s I autocorrelation tests between the distance of sampling sites of the biotic-dispersed tree seedling community and the residuals of the linear models between α- and β-diversity metrics (*^0^D* = all species, *^1^D* = typical species and *^2^D* = dominant species) and landscape metrics in a fragmented tropical forest in southeastern Mexico. The numbers in parenthesis indicate de standard deviation. The subscript numbers indicate the scale of effect of each variable.

| Diversity | Order | Landscape metric | Moran’s I | *P* value |
| --- | --- | --- | --- | --- |
| α | ***^0^α*** | AI_600_ | -0.036 (0.064) | 0.632 |
|  |  | SF_600_ | -0.064 (0.066) | 0.967 |
|  |  | PI_1200_ | -0.067 (0.065) | 0.999 |
|  | ***^1^α*** | AI_600_ | -0.045 (0.064) | 0.74 |
|  |  | SF_600_ | -0.1 (0.065) | 0.61 |
|  |  | PI_1200_ | -0.032 (0.064) | 0.588 |
|  | ***^2^α*** | A_I600_ | -0.057 (0.064) | 0.877 |
|  |  | SF_600_ | -0.096 (0.063) | 0.643 |
|  |  | PI_1200_ | -0.023 (0.065) | 0.496 |
| β | ***^0^β*** | EC_800_ | -0.134 (0.057) | 0.24 |
|  |  | PD_1400_ | -0.055 (0.053) | 0.827 |
|  | ***^1^β*** | AI_500_ | -0.073 (0.065) | 0.922 |
|  |  | SF_1300_ | -0.164 (0.064) | 0.129 |
|  | ***^2^β*** | AI_500_ | -0.123 (0.064) | 0.383 |
|  |  | PD_500_ | -0.173 (0.064) | 0.098 |

Table H. Tree seedlings recorded during the sampling period in the Lacandona rainforest, at southeastern Mexico. Seedling dispersal syndromes were categorized whether by abiotic (A) or biotic (B) dispersion.

| *Family* | Genus | Species | Dispersal | Number |
| --- | --- | --- | --- | --- |
| ACANTHACEAE | *Bravaisia* | *B. integerrima* | A | 29 |
| ANACARDIACEAE | *Spondias* | *S. mombin* | B | 6 |
|  |  | *S. radlkoferi* | B | 4 |
|  | *Astronium* | *A. graveolens* | B | 1 |
| ANNONACEAE | *Annona* | *A. sp1* | B | 4 |
|  | *Xylopia* | *X. frutescens* | B | 1 |
| APOCYNACEAE | *Stemmadenia* | *S. galeottiana* | B | 2 |
| BIGNONIACEAE | *Tabebuia* | *T. rosea* | A | 1 |
| BORAGINACEAE | *Cordia* | *C. odorata* | B | 4 |
|  |  | *C. bicolor* | B | 2 |
| BURSERACEAE | *Bursera* | *B. simaruba* | B | 1 |
|  | *Protium* | *P. copal* | B | 1 |
| CAPPARACEAE | *Capparis* | *C. quiriguensis* | B | 1 |
| CHRYSOBALANACEAE | *Hirtella* | *H. americana* | B | 15 |
|  | *Licania* | *L. hypoleuca* | B | 8 |
|  |  | *L. platypus* | B | 1 |
| CLUSIACEAE | *Calophyllum* | *C. brasilense* | B | 10 |
|  | *Garcinia* | *G. intermedia* | B | 4 |
| EUPHORBIACEAE | *Croton* | *C. schiedeanus* | B | 11 |
| FABACEAE | *Acacia* | *A. mayana* | B | 11 |
|  | *Albizia* | *A. leucocalyx* | A | 5 |
|  | *Andira* | *A. inermis* | B | 4 |
|  | *Cojoba* | *C. arboreum* | B | 4 |
|  | *Dialium* | *D. guianense* | B | 10 |
|  | *Inga* | *I. edulis* | B | 3 |
|  |  | *I. pavoniana* | B | 1 |
|  |  | *I. punctata* | B | 418 |
|  |  | *I. vera* | B | 2 |
|  |  | *I. sp1* | B | 4 |
|  |  | *I. sp2* | B | 2 |
|  | *Lonchocarpus* | *L. cruentus* | A | 2 |
|  |  | *L. pubescens* | A | 8 |
|  | *Platymiscium* | *P. yucatanum* | A | 10 |
|  | *Pterocarpus* | *P. hayesii* | A | 1 |
|  |  | *P. yucatanum* | A | 1 |
| ICACINACEAE | *Calatola* | *C. costaricanum* | B | 3 |
| LASCISTEMATACEAE | *Lacistema* | *L. agreggatum* | B | 2 |
| LAURACEAE | *Licaria* | *L. capitata* | B | 8 |
|  | *Nectandra* | *N. ambigiens* | B | 17 |
|  |  | *N. reticulata* | B | 5 |
| MALVACEAE | *Hampea* | *H. stipitata* | B | 4 |
|  | *Luehea* | *L. seemannii* | A | 4 |
|  | *Quararibea* | *Q. funebris* | B | 4 |
| MELIACEAE | *Cedrela* | *C. odorata* | A | 3 |
|  | *Guarea* | *G. excelsa* | B | 8 |
|  |  | *G. glabra* | B | 9 |
|  |  | *G. grandifolia* | B | 5 |
|  | *Trichilia* | *T. havanensis* | B | 3 |
| MORACEAE | *Brosimum* | *B. alicastrum* | B | 147 |
|  |  | *B. latescens* | B | 20 |
|  | *Castilla* | *Castilla elastica* | B | 32 |
|  | *Maclura* | *M. tinctoria* | B | 3 |
|  | *Trophis* | *T. racemosa* | B | 93 |
| PRIMULACEAE | *Ardisia* | *A. paschalis* | B | 27 |
| MYRTACEAE | *Eugenia* | *E. edulis* | B | 4 |
|  |  | *E. mexicana* | B | 57 |
|  |  | *E. nigrita* | B | 5 |
| POLYGONACEAE | *Coccoloba* | *C. barbadensis* | B | 1 |
| RUBIACEAE | *Faramea* | *F. occidentalis* | B | 20 |
|  | *Posoqueria* | *P. latifolia* | B | 13 |
|  |  | *P. sp1* | B | 5 |
| SALICACEAE | *Pleuranthodendron* | *P. lindenii* | B | 18 |
| SAPINDACEAE | *Cupania* | *C. dentata* | B | 8 |
|  |  | *C. glabra* | B | 14 |
|  | *Sapindus* | *S. saponaria* | B | 1 |
| SAPOTACEAE | *Chrysophyllum* | *C. mexicanum* | B | 10 |
|  | *Pouteria* | *P. durlandii* | B | 4 |
| ULMACEAE | *Ampelocera* | *A. hottlei* | B | 173 |
| VOCHYSIACEAE | *Vochysia* | *V. guatemalensis* | A | 7 |
| Unknown |  | Morpho 1 |  | 1 |
|  |  | Morpho 2 |  | 3 |
|  |  | Morpho 3 |  | 1 |

Table I. Model selection results of the linear models that explain each α- and β-diversity metric (*^0^D* = all species, *^1^D* = typical species and *^2^D* = dominant species) of the animal-dispersed tree seedling community in a fragmented tropical rainforest at southern Mexico. The models log-likelihood (*LL*), the corrected Akaike index criterion (*AICc*), the Akaike difference from the best model (*Δ*), the Akaike weight (*w_i_*) and the coefficient of determination (*R^2^*). The landscape metrics are the aggregation index (AI), the edge contrast index (EC), patch isolation (PI), and the percentage of secondary forest (SF). The subscript numbers indicate the scale of effect (radius meters) of each landscape metric. The superscript symbols indicate the positive (+) or negative (-) effects of landscape metrics. The values in italic correspond to the null model.

|  | Diversity metric | Model | *R^2^* | *LL* | *AICc* | *Δ* | *w_i_* |
| --- | --- | --- | --- | --- | --- | --- | --- |
| α-diversity | All species | ^0^α ~ ^+^AI_600_ + ^-^SF_600_ | 0.6 | -8.38 | 28.39 | 0 | 0.52 |
|  |  | ^0^α ~ ^-^SF_600_ | 0.45 | -11.46 | 30.91 | 2.52 | 0.15 |
|  |  | ^0^α ~ ^-^PI_1200_ + ^-^SF_600_ | 0.52 | -9.87 | 31.38 | 2.98 | 0.12 |
|  |  | ^0^α ~ ^+^AI_600_ + ^-^PI_1200_ + ^-^SF_600_ | 0.6 | -7.69 | 31.39 | 2.99 | 0.12 |
|  |  | ^0^α ~ ^+^AI_600_ | 0.39 | -12.33 | 32.66 | 4.27 | 0.06 |
|  |  | ^0^α ~ ^+^AI_600_ + ^-^PI_1200_ | 0.43 | -11.25 | 34.13 | 5.74 | 0.03 |
|  |  | ^0^α ~ ^-^PI_1200_ | 0.24 | -14.1 | 36.21 | 7.82 | 0.01 |
|  |  | ^0^α ~ 1 | *0* | *-16.84* | *38.61* | *10.22* | *0* |
|  | Typical species |  |  |  |  |  |  |
|  |  | ^1^α ~ ^-^PI_1200_ + ^-^SF_600_ | 0.67 | -1.84 | 15.31 | 0 | 0.32 |
|  |  | ^1^α ~ ^+^AI_600_ + ^-^SF_600_ | 0.67 | -1.93 | 15.5 | 0.19 | 0.29 |
|  |  | ^1^α ~ ^-^SF_600_ | 0.61 | -3.81 | 15.63 | 0.32 | 0.27 |
|  |  | ^1^α ~ ^+^AI_600_ + ^-^PI_1200_ + ^-^SF_600_ | 0.69 | -0.78 | 17.57 | 2.26 | 0.1 |
|  |  | ^1^α ~ ^+^AI_600_ | 0.29 | -8.64 | 25.29 | 9.98 | 0 |
|  |  | ^1^α ~ ^-^PI_1200_ | 0.26 | -8.99 | 25.98 | 10.67 | 0 |
|  |  | ^1^α ~ ^+^AI_600_ + ^-^PI_1200_ | 0.36 | -7.25 | 26.13 | 10.82 | 0 |
|  |  | ^1^α ~ 1 | *0* | *-11.94* | *28.8* | *13.49* | *0* |
|  | Dominant species |  |  |  |  |  |  |
|  |  | ^2^α ~ ^-^PI_1200_ + ^-^SF_600_ | 0.74 | 2.42 | 6.8 | 0 | 0.49 |
|  |  | ^2^α ~ ^-^SF_600_ | 0.67 | 0.03 | 7.95 | 1.14 | 0.28 |
|  |  | ^2^α ~ ^+^AI_600_ + ^-^SF_600_ | 0.7 | 1.23 | 9.17 | 2.37 | 0.15 |
|  |  | ^2^α ~ ^+^AI_600_ + ^-^PI_1200_ + ^-^SF_600_ | 0.73 | 2.88 | 10.23 | 3.43 | 0.09 |
|  |  | ^2^α ~ ^-^PI_1200_ | 0.27 | -6.39 | 20.79 | 13.99 | 0 |
|  |  | ^2^α ~ ^+^AI_600_ | 0.23 | -6.83 | 21.65 | 14.85 | 0 |
|  |  | ^2^α ~ ^+^AI_600_ + ^-^PI_1200_ | 0.33 | -5.19 | 22.02 | 15.22 | 0 |
|  |  | ^2^α ~ 1 | *0* | *-9.49* | *23.91* | *17.11* | *0* |
| β-diversity | All species |  |  |  |  |  |  |
|  |  | ^0^β ~ ^-^EC_800_ | 0.4 | -11.04 | 30.08 | 0 | 0.56 |
|  |  | ^0^β ~ ^-^EC_800_ + ^+^PD_1400_ | 0.45 | -9.77 | 31.17 | 1.08 | 0.32 |
|  |  | ^0^β ~ ^+^PD_1400_ | 0.25 | -12.79 | 33.59 | 3.5 | 0.1 |
|  |  | ^0^β ~ 1 | *0* | *-15.68* | *36.27* | *6.19* | *0.03* |
|  | Typical species |  |  |  |  |  |  |
|  |  | ^1^β ~ ^+^PD_500_ | 0.44 | -13.71 | 35.41 | 0 | 0.63 |
|  |  | ^1^β ~ ^+^PD_500_ + ^-^SF_1300_ | 0.48 | -12.61 | 36.86 | 1.44 | 0.3 |
|  |  | ^1^β ~ ^-^SF_1300_ | 0.24 | -16.19 | 40.37 | 4.96 | 0.05 |
|  |  | ^1^β ~ 1 | *0* | *-18.94* | *42.8* | *7.39* | *0.02* |
|  | Dominant species |  |  |  |  |  |  |
|  |  | ^2^β ~ ^+^PD_500_ + ^-^SF_1300_ | 0.45 | -9.81 | 27.62 | 0 | 0.56 |
|  |  | ^2^β ~ ^-^AI_500_ + ^+^PD_500_ + ^-^SF_1300_ | 0.49 | -8.6 | 28.84 | 1.23 | 0.3 |
|  |  | ^2^β ~ ^-^AI_500_ + ^-^SF_1300_ | 0.34 | -11.32 | 30.64 | 3.02 | 0.12 |
|  |  | ^2^β ~ 1 | *0* | *-15.17* | *35.26* | *7.64* | *0.01* |

Figure A. Association between buffer size and the coefficient of prediction (*R^2^_CV_*) between each landscape metric and each α-diversity metric (all, typical and dominant species) of the animal-dispersed tree seedling community in a fragmented rainforest in southeastern Mexico. Each point represents the *R^2^_CV_* value of a simple linear regression between a diversity metric and single landscape metric measured at each buffer size. The points above the horizontal grey line indicate the proportion of variation that can be predicted by the model, whereas those points below the horizontal line indicate the prediction power of the model is worse than for a null model. The scale of effect is indicated within each panel with vertical lines.


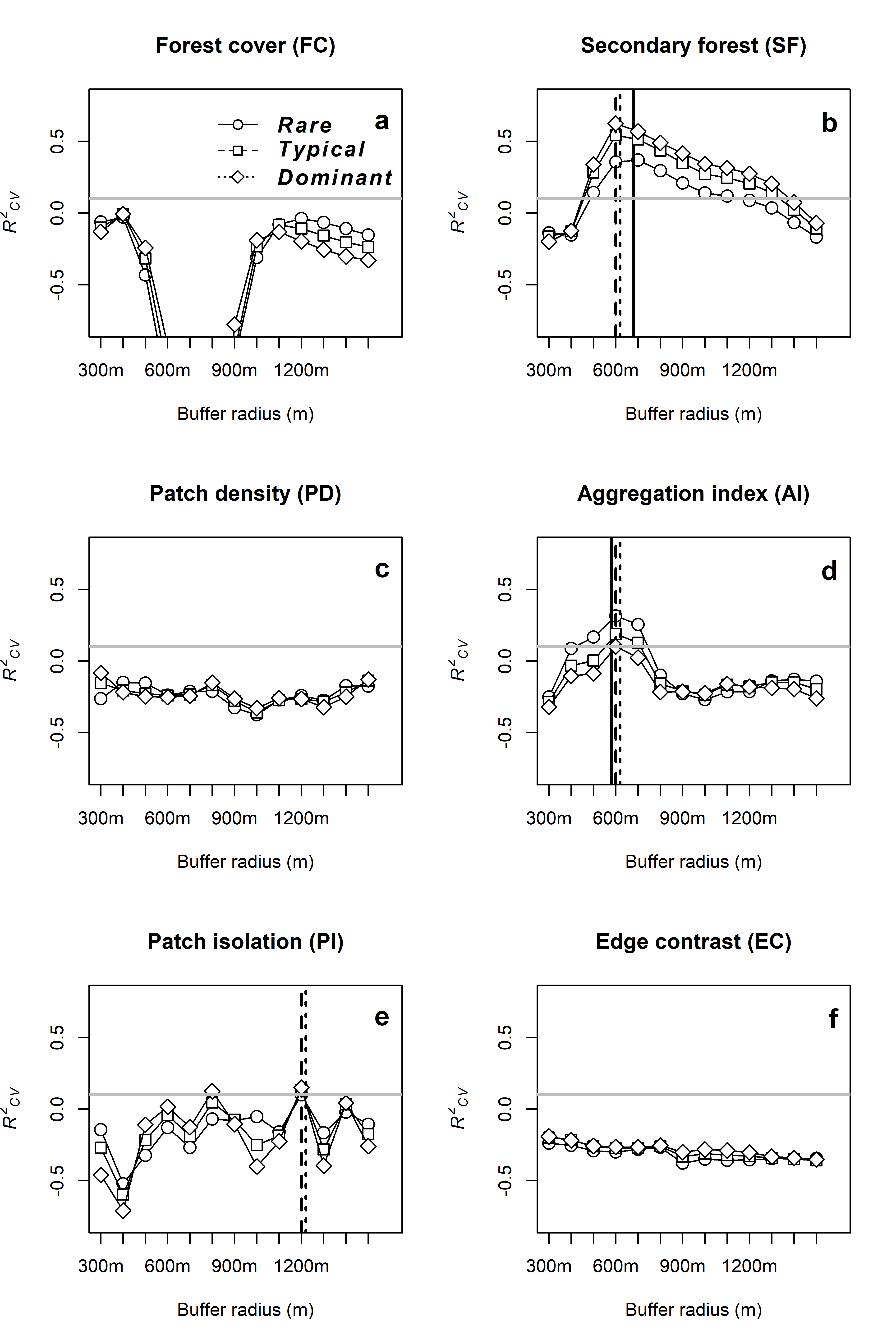


Figure B. Association between buffer size and the coefficient of prediction (*R^2^_CV_*) between each landscape metric and each β-diversity metric (all, typical and dominant species) of the animal-dispersed tree seedling community in a fragmented rainforest in southeastern Mexico. Each point represents the *R^2^_CV_* value of a simple linear regression between a diversity metric and single landscape metric measured at each buffer size. The points above the horizontal grey line indicate the proportion of variation that can be predicted by the model, whereas those points below the horizontal line indicate the prediction power of the model is worse than for a null model. The scale of effect is indicated within each panel with vertical lines.


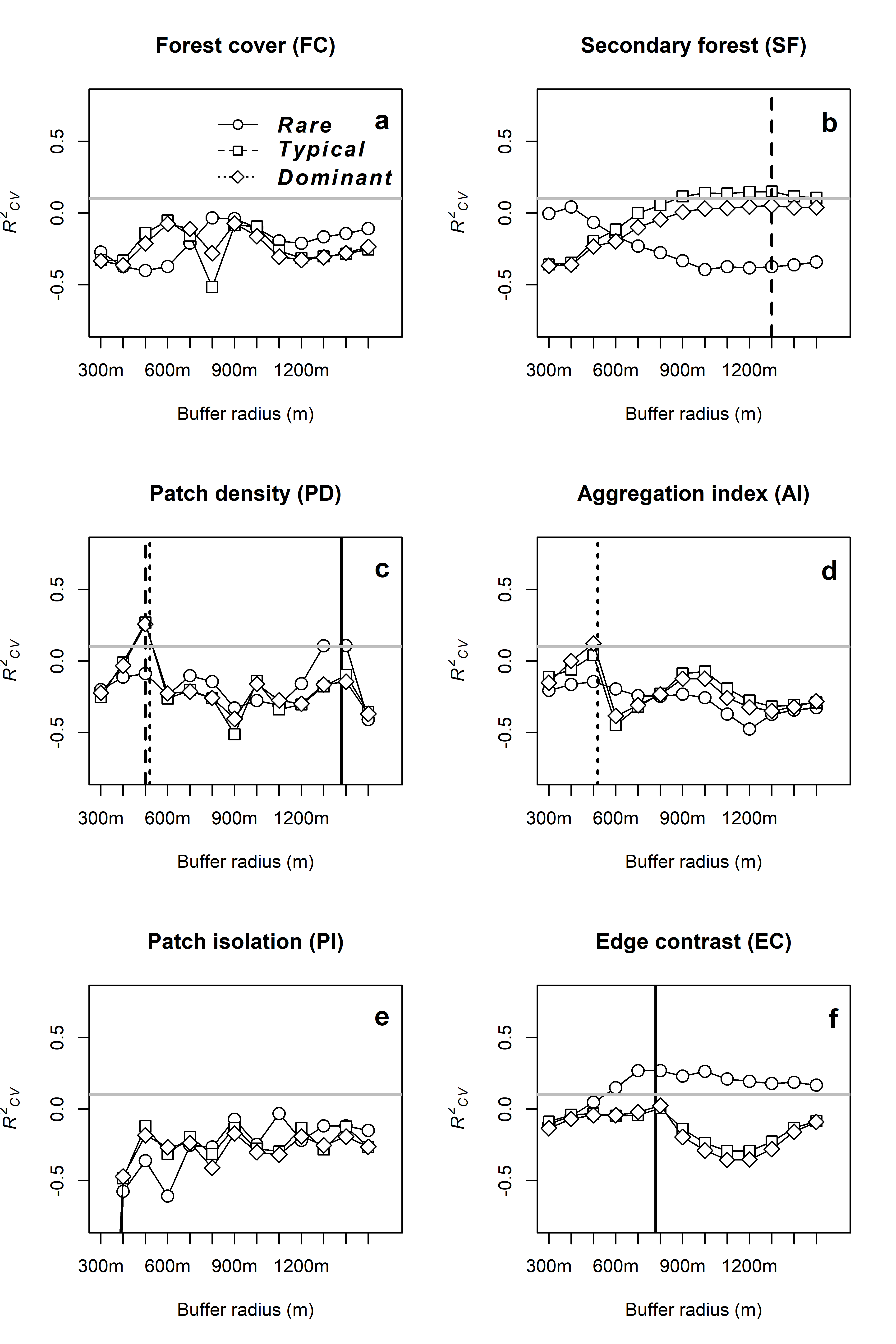


Figure C. Uncertainty around the estimated scale of effect between each landscape metric and each α-diversity metric (all, typical and dominant species) of the animal-dispersed tree seedling community, estimated by bootstrapping. For each landscape-diversity combination we randomly re-sampled the data from *n* patches, with replacement, from the set of the 16 forest patches, 1000 times. We then estimated the scale of effect for each resampled data. The total number of times (out of 1000) that each buffer size was selected as the scale of effect is plotted. The estimated scale of effect (red arrow) is the buffer size with the highest *R^2^_CV_* for the complete data set (see Fig A).


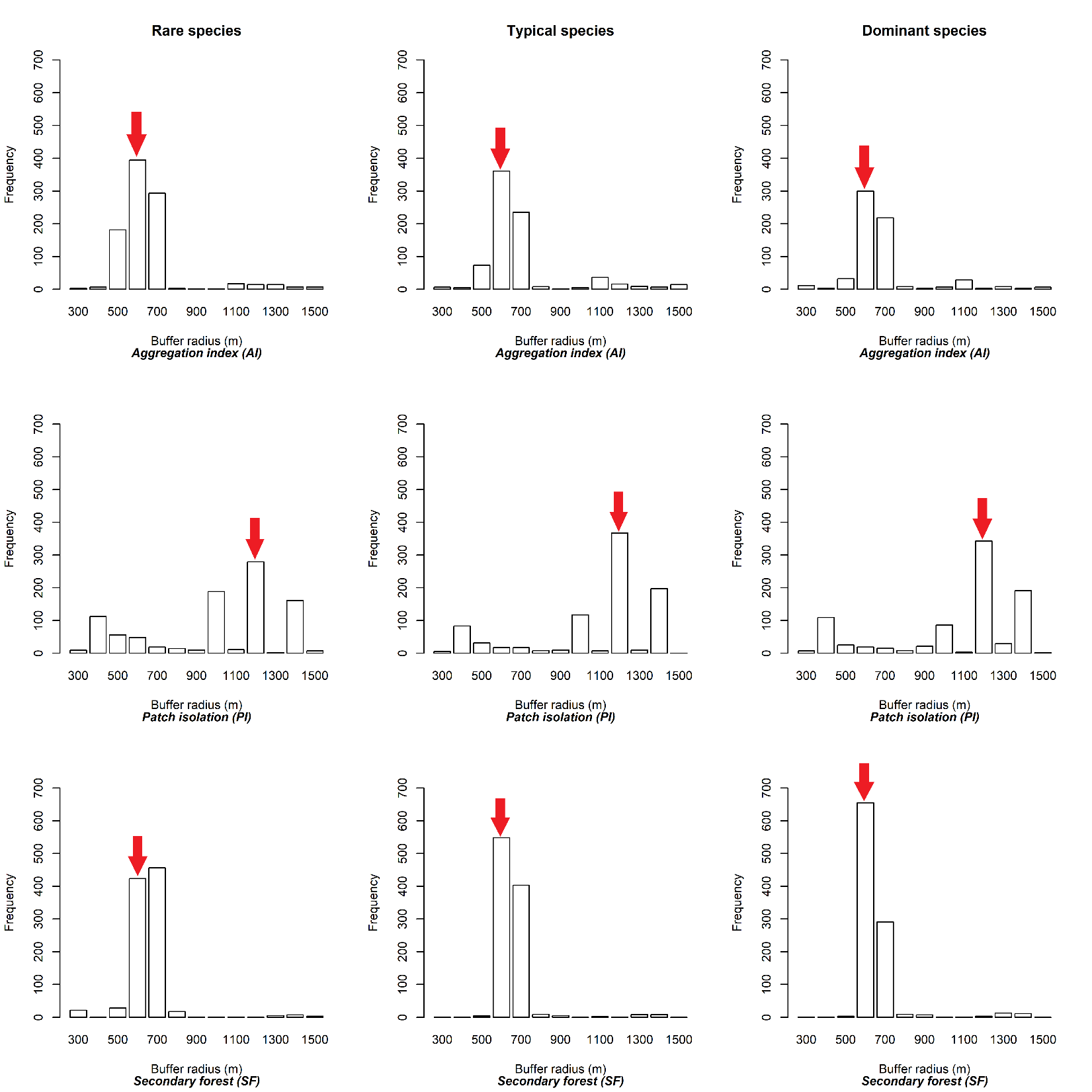


Figure D. Uncertainty around the estimated scale of effect between each landscape metric and each β-diversity metric (all, typical and dominant species) of the animal-dispersed tree seedling community, estimated by bootstrapping. For each landscape-diversity combination we randomly re-sampled the data from *n* patches, with replacement, from the set of the 16 forest patches, 1000 times. We then estimated the scale of effect for each resampled data. The total number of times (out of 1000) that each buffer size was selected as the scale of effect is plotted. The estimated scale of effect (red arrow) is the buffer size with the highest *R^2^_CV_* for the complete data set (see Fig B).


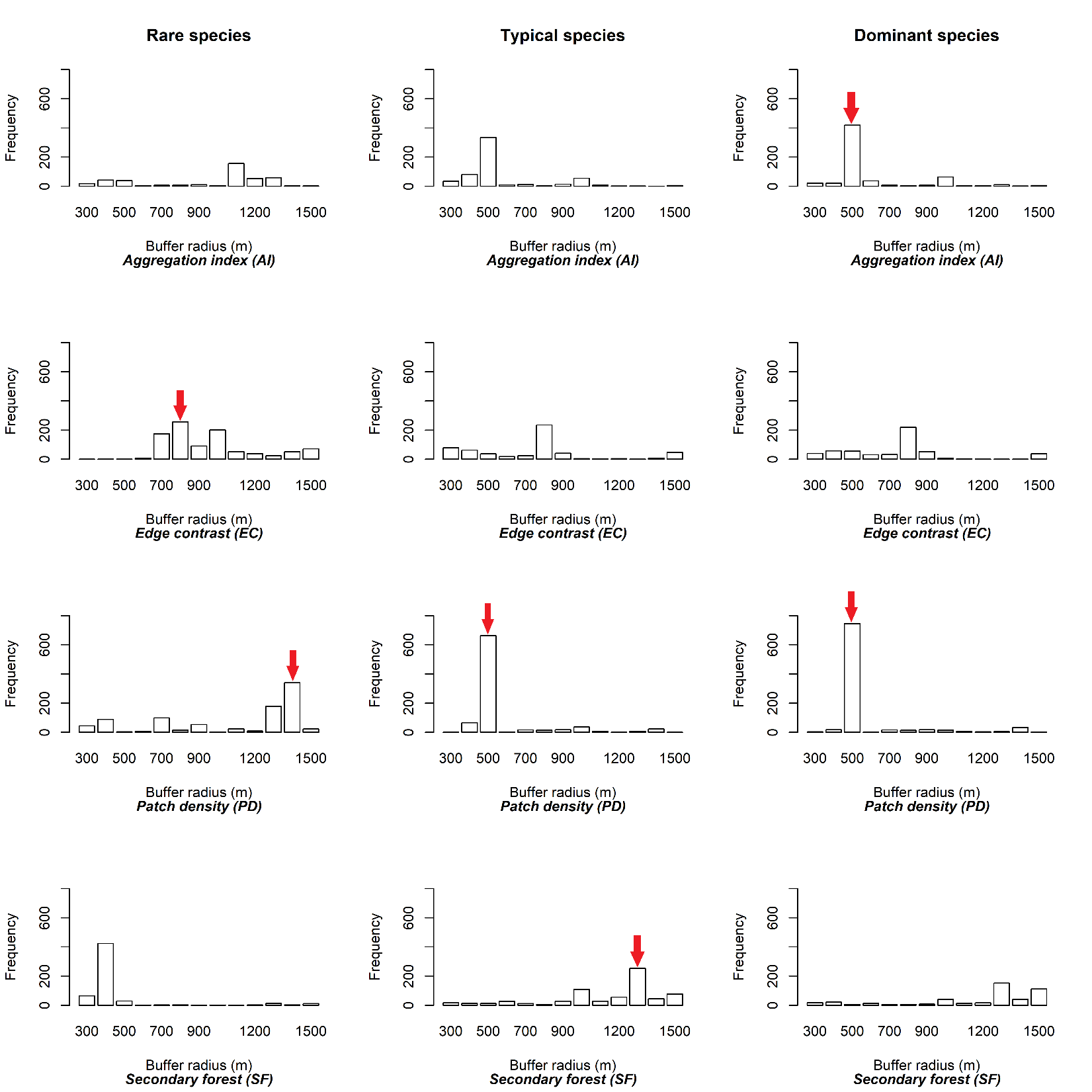


Figure E. Values of a) α- and b) β-diversity orders for tree seedlings within the patches’ sampling plots in a fragmented tropical forest in southeastern Mexico. We observed ^0^α-diversity was significantly higher (2.703 ± 0.716) than ^1^α (2.295 ± 0.527) and ^2^α (2.114 ± 0.452) within sampling plots (χ^2^ = 6.99; *d.f.* = 2; *P* = 0.0302). This also held true for β-diversity (χ^2^ = 32.511; *d.f.* = 2; *P* < 0.001; Figure 2b), which had ^0^β values higher (4.303 ± 0.665) than ^1^β (2.258 ± 0.816) and ^2^β (1.617 ± 0.644).


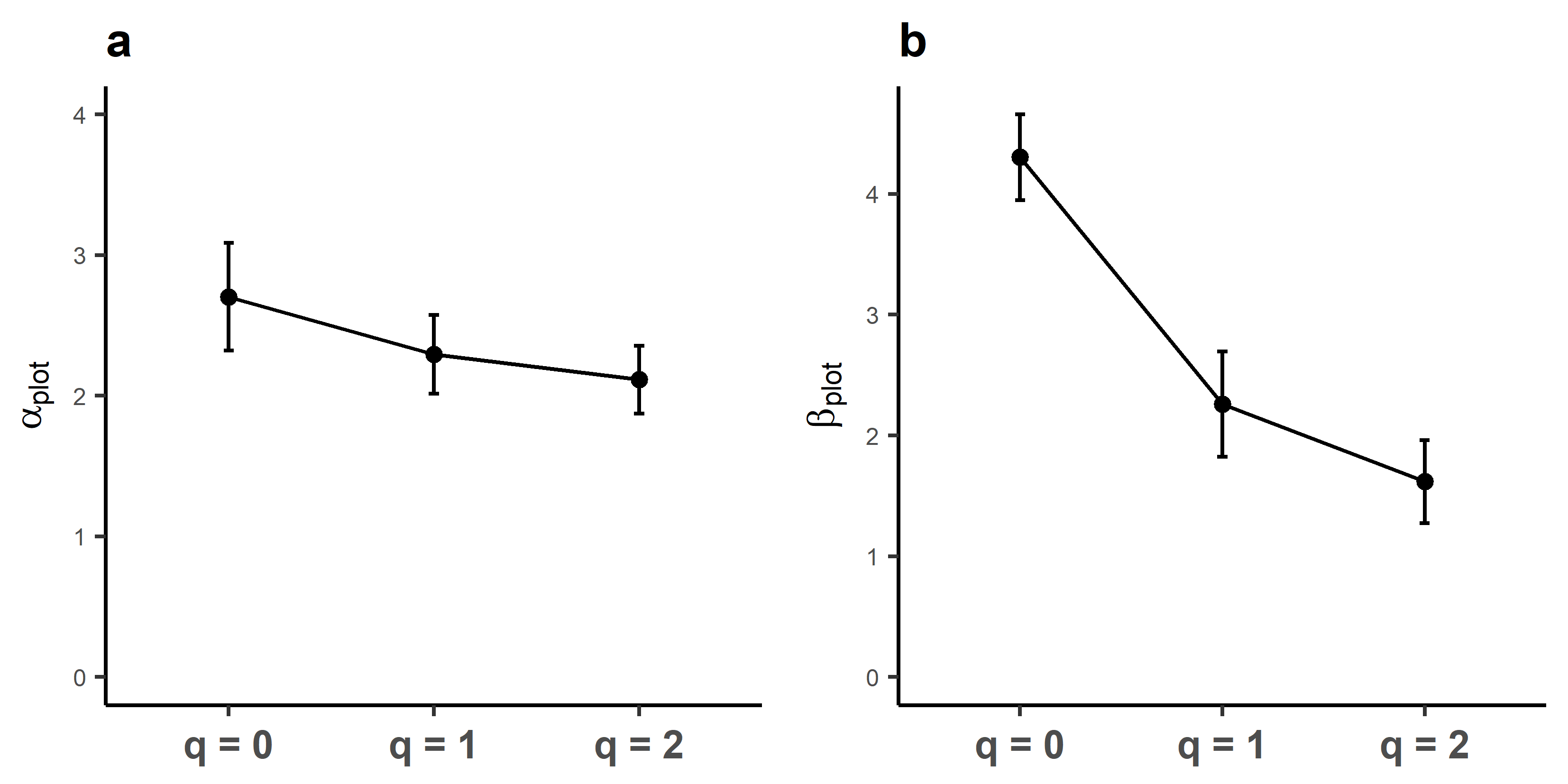


Figure F. Differences in the scale of effect between each landscape metric and each α- and β-diversity metric of the animal-dispersed tree seedling community. The values employed were estimated by bootstrapping. For each landscape-diversity combination we randomly re-sampled the data from *n* patches, with replacement, from the set of the 16 forest patches, 1000 times. We then estimated the scale of effect for each resampled data. The bars represent the mean and the whiskers the standard error.


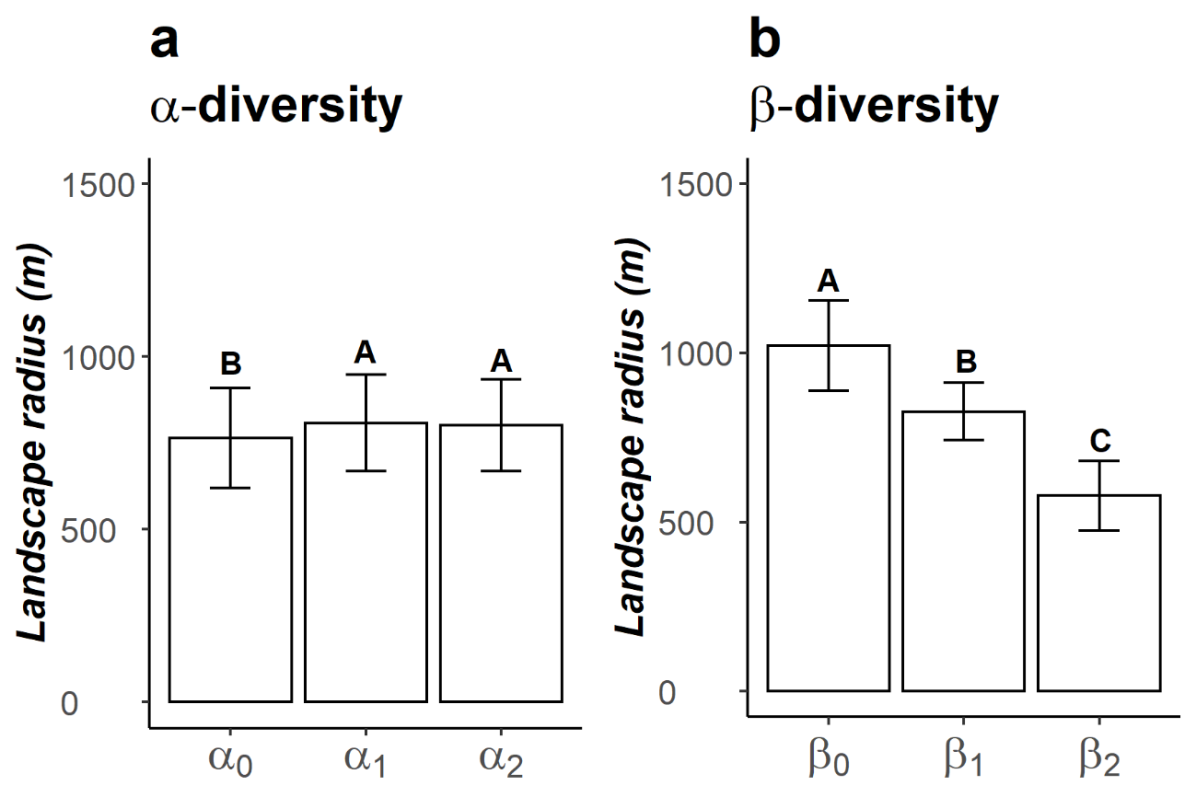

Supplement: S1 Text — (DOCX) [file pone.0253284.s001.docx]
