# Supplementary material for: Landscape structure shapes the diversity of tree seedlings at multiple spatial scales in a fragmented tropical rainforest
Source: PLoS One. 2021 Jul 16;16(7):e0253284. doi: 10.1371/journal.pone.0253284 (PMC8284835; doi:10.1371/journal.pone.0253284)
Supplement: Attachment — Submitted filename: Response to Reviewers.docx [file pone.0253284.s002.docx]

Dr. Juliana Hipólito

Academic Editor

PlosOne

Dear Dr Hipólito,

Please find enclosed the reviewed version of the manuscript PONE-D-19-30178R1, now entitled “**Landscape structure shapes the diversity of tree seedlings at multiple spatial scales in a fragmented tropical rainforest**”. The author and co-authors are grateful for the constructive and detailed reviews of the Academic Editor and three anonymous reviewers. We appreciate the valuable comments and dedication of the reviewers.

We are confident that we have adequately incorporated all the reviewers´ suggestions and critical comments, which have clearly benefited the manuscript. We included all the assessments regarding the spatial autocorrelation among response variables, landscape metrics and models residuals in the Supplementary Information. We also included the assessment of the uncertainty around the scale of effect. All minor edits were also corrected. Below you will find a detailed response to each of the observations raised by the reviewers.

Please, note that to avoid redundancy in our responses, we organized all comments and our responses by sections and topics. We also indicate the reviewer’s name at the beginning of each comment (1 or 2).

We thank you again for the opportunity that our study is considered for publication in PlosOne. We are looking forward to receiving your decision

Yours sincerely,

Sergio Nicasio-Arzeta (on behalf of my co-authors)

EDITOR: Thank you for submitting your manuscript to PLOS ONE. After careful consideration, we feel that it has merit but does not fully meet PLOS ONE’s publication criteria as it currently stands. Therefore, we invite you to submit a revised version of the manuscript that addresses the points raised during the review process.

***Authors: We are grateful for the careful review performed by the reviewers and yourself***

REVIEWER COMMENTS

-------------------------------------GENERAL COMMENTS--------------------------------------

REVIEWER 1: The article improved and is accessible to a broader audience now. The authors corrected some mistakes without changing the main results. All my suggestions and questions were adequately addressed.

REVIEWER 2: I congratulate the authors for this extremely relevant work that will certainly represent an important contribution to the field. In this study the authors investigate the effect of habitat loss and fragmentation on the diversity of tree seedlings diversity in tropical forests in Mexico.

***Authors: Thank you very much for this positive feedback***

----------------------------------------------ABSTRACT-------------------------------------------------

REVIEWER 2: Line 24: What are “biotically dispersed”? Are you referring just to zoochory or other modalities of dispersal mediated by biologic processes such as ballistic dispersal are also included? I suggest you be more specific.

***Authors: We changed it by “animal-dispersed tree species”***

REVIEWER 1: Lines 30 to 32: Consider to rephrase as: " We found that α-diversity was positively affected by patch aggregation and negatively affected by the amount of surrounding secondary forests."

***Authors: Corrected as suggested***

-------------------------------------------INTRODUCTION--------------------------------------------

REVIEWER 2: Despite the wheel gathered data and the clear improvements from the previews versions there are a few issues that undermines the good work done and if addressed certainly will improve the quality of the manuscript to the level required for publication in PLOS ONE. Is with the spirit to contribute to this processes that I make some remarks and suggestions. The logical and theoretical structure of this manuscript needs some attention. The authors adopted a hierarchical approach to their study, however failed in clearly explain the causal mechanisms linking the landscape pattern and the seedlings α and β diversities as they were measured in the field. There are some issues with the unusual way that the α and β diversities were defined and measured in this study that are in the root of this confusion. This problem was somewhat pointed by other reviewers in previous versions however despite the substantial improvements, some inconsistencies regarding the relation between the causal mechanisms described in the introduction, the methods adopted, the results and discussion remain. This are also reflected in the stated objectives. They were poorly postulated and are not properly justified based on the arguments presented in the introduction. Whereas the methods adopted in this study were adequate to answer the most general question, “Which components of landscape structure are influencing seedling α- and β-diversity and at what spatial scales? they were insufficient to evaluate the specific the hypothesis stated in 119 – 124. In addition, apparently there are some confusion through the manuscript to clarify what are the explanandum and explanans of the study. They must make clear that the explanandum must is the seedlings diversity and the explanans the landscape structure, whereas the seed dispersers are only part of the accessory argument necessary to establish a causal link between those two since there was no direct assessment of it. Because this confusion the introduction generates the expectative that the study would also investigate the relationship between the landscape structure and the seed dispersers and predation and that those are the explanans of the seedling’s diversity. This confusion permeates to the conclusions were the authors make assertions about the demonstrated effect of the landscape structure over the seed dispersers without making any indication that they didn’t observed them directly.

***Authors: We have rewritten the Introduction accordingly and now emphasized how landscape structure is associated with seedling a- and b-diversity. We clarified that seedling diversity is the explanandum in lines 58-70 and that landscape composition and configuration are the explanans in lines 71-93. Attending these recommendations, we emphasized that we did not make observations on animal seed dispersers, as well as properly justified our specific hypothesis in line 122-125. For further details, please, see below.***

REVIEWER 2: Lines 51 – 52: What “ecological function”? For clarity I suggest that you speak about a specific function or just refer to biodiversity maintenance.

***Authors: Changed to “It is well known that resources of plants and animals within forest patches rely on the diversity and structure of the tree community [7,13,14], which in turn would be determined by the species richness (α-diversity) and compositional similarity (β-diversity) of the seedling community [15,16]” (lines 58-61).***

REVIEWER 1: Lines 58 - 60: In "Evidence from fragmented rainforest indicates that forest loss and fragmentation limit the inter-patch movement of arboreal vertebrates [9], and consequently, the α- diversity and abundance of biotically dispersed and large-sized seeds of old-growth tree species [10]." Why would inter-patch movements increase a-diversity and abundance of seedlings? Because they would bring large seeds from the other patch? Because patch isolation would lead to the local absence of arboreal vertebrates, reducing seed dispersal of old-growth tree species? Or both?

***Authors: We changed the sentence as follow: “On the one hand, the α-diversity decline of animal-dispersed seeds by forest loss [35,36], suggests that landscape composition may have strong effects on seedling α-diversity by limiting seed dispersal among patches.” (lines 75-77).***

***The inter-patch movement in fragmented habitats plays a key role in the persistence of seed dispersers within patches. Birds and arboreal primates are the most important seed dispersers of the old-growth tree species in tropical rainforests. Our sentence change emphasizes that seed dispersal is associated to landscape composition, particularly the percentage of old-growth forest in the landscape. Recent studies have shown that forest loss is a key factor in the species richness decline of animal-dispersed seeds across the landscape (San-José et al. 2019a; San-José et al. 2019b). The inter-patch distance increases as the percentage of forest decreases (Fahrig, 2003), limiting the inter-patch movement and leading to the local absence of dispersal vectors, such as birds and arboreal primates (Carrara et al., 2015; Galán-Acedo et al., 2018; Galán-Acedo et al., 2019).***

REVIEWER 1: Line 63 - "at plot level" - I appreciated the inclusion of this explanation; however, the description of the "plot level" will appear later in the text. I suggest using something like "between sites in a forest fragment" or "B-diversity within a forest fragment" or another expression that you prefer.

***Authors: Changed as “Since these animals play a key role in the composition of seedling communities [25,41,42], edge contrast may be affecting β-diversity of seedlings by limiting or promoting the presence of seed dispersers, seed predators and herbivorous mammals.” (lines 90-93).***

REVIEWER 1: Line 63 - "regulate seedling recruitment": How do mammals regulate? Do they increase or decrease seedling recruitment of large-seeded tree species?

***Authors: Frugivore activity may influence the abundance and richness of seedling banks throughout seed removal and dispersal, and seedling herbivory and trampling.***

REVIEWER 2: Lines 62 – 64: Here the confusion about diversity partitioning begins. Why there is a reference to plot diversity in the introduction? Plot does not have meaning without a specific description. However, I think you do not need to talk about plots this early. Instead you need to find another way to explain to the readers wat the levels of analysis represent in biological terms, then when you explain the rationale behind the sampling methods it will be easier to understand to link with the biological sense.

***Authors: Changed as “Since these animals play a key role in the composition of seedling communities [25,41,42], edge contrast may be affecting β-diversity of seedlings by limiting or promoting the presence of seed dispersers, seed predators and herbivorous mammals.” (lines 90-93).***

REVIEWER 2: Line 65: Again, function without contextualization does not have a clear meaning. You need to be more specific. To what function are you referring to?

***Authors: We deleted the line 65 to avoid misinterpretations.***

REVIEWER 2: Lines 68 – 70: Besides the simplicity of the meaning that it is communicating, this sentence is hard to read as it is. For clarity you should rewrite it.

***Authors: We rewrote it as “On the one hand, the α-diversity decline of animal-dispersed seeds by forest loss [35,36], suggests that landscape composition may have strong effects on seedling α-diversity by limiting seed dispersal among patches” (lines 75-77).***

REVIEWER 1: Lines 70-71: Consider to substitute "These landscape metrics" by "These landscape attributes" or These landscape characteristics" - metrics are measurements to represent those structural characteristics.

***Authors: Corrected as “landscape composition” (line 76)***

REVIEWER 1: Line 71: promote seed dispersal by vertebrates at long-distances". Would it be between or within patches?

***Authors: It is among patches. We mentioned that to avoid confusion in line 77.***

REVIEWER 2: Lines 70 – 72: What landscape metrics? How a landscape metric promote dispersal? You are confounding the conceptual quantity with the measurement. One thing is the “spatial proximity”, the other thing is Euclidian Nearest Neighbor measured on a binary map.

***Authors: We corrected by deleting “landscape metrics” and using only “landscape composition” (line 77) to avoid further confusions.***

REVIEWER 2: Lines 72 – 73: This sentence is very confusing. Do you mean that the spatial proximity among forest patches is more crucial in highly deforested landscapes? How much is highly deforested? This sentence is true to a landscape with 95% deforestation or in that scenario it is too late? For clarity I suggest you rewrite this sentence. In addition, how old is “old-growth”?

***Authors: We corrected as “On the one hand, the α-diversity decline of animal-dispersed seeds by forest loss [35,36], suggests that landscape composition may have strong effects on seedling α-diversity by limiting seed dispersal among patches” (lines 75-77). We focused on the role of spatial proximity in highly deforested landscapes in the Discussion section. Also, we limited the term “old-growth” for tree species attributes. Instead, we referred to the remaining mature forest in the landscape as “forest cover”.***

REVIEWER 1: Line 74 - Consider to write "within patch B-diversity".

***Authors: Corrected as suggested (line 85)***

REVIEWER 2: Line 74: I did not understand why you used the adverb “Conversely” to connect the paragraphs and I don’t think there is a coma in “tree seedlings, is more sensitive”.

***Authors: Corrected as “On the other hand…” (line 81)***

REVIEWER 2: Lines 74 – 75: This is a confusing sentence… What β-diversity? The diversity among forest patches? The problem with α, β and γ diversities is that they are very context dependent. You need to describe the hierarchical structure of your rationale before talking about α, β and γ diversities, otherwise it will necessarily very confusing, especially in your case were the use of these terms is atypical. In addition, how the abundance of seedlings dives β-diversity? Are you referring to the dominance of pioneer seedlings? If so, it is not clear… one thing is seedlings abundance, other thing is the dominance of a specific kind of seedling.

***Authors: We corrected to emphasize that β-diversity changes resulted from species turnover. Additionally, we stated that we expect β-diversity changes within forest patches (among plots). Therefore, we refer to α-diversity as the average number of species within plots, whereas γ-diversity is the total number of species within each patch.***

***Thus, we corrected as “On the other hand, seedling β-diversity within patches may be linked to landscape configuration patterns of forest fragmentation and edge effects. Studies on tree communities have found that dispersal limitation by forest fragmentation might be involved in the reduction of shared tree species within forest patches, promoting β-diversity within and among patches [34]” (lines 81-85).***

REVIEWER 2: Lines 81 - 84: This is a very confusing statement… How the “matrix” vegetation is similar with the forest? Do you mean that, when the dispensers are present there are more occurrences of species characteristic of old-growth forest?

***Authors: Matrix similarity with forest cover refers to the structural resemblance of matrix vegetation to the mature vegetation of rainforests. This assumption indicates that secondary forests have a greater structural resemblance to mature forest than cattle pastures. Such resemblance provides of better habitat conditions for terrestrial mammals, which can use these matrix covers as complementary/supplementary resources or as safe corridors for the inter-patch movement.***

***We understood the confusions derived from these lines. Therefore, we corrected as “Edge contrast is also involved in the inter-patch movement that sustain the persistence of animal seed dispersers and herbivorous mammals within patches [6,40]. Since these animals play a key role in the composition of seedling communities [25,41,42], edge contrast may be affecting β-diversity of seedlings by whether limiting or promoting the presence of seed dispersers, seed predators and herbivorous mammals.” (lines 90-93)***

REVIEWER 2: Lines 87 – 88: And the confusion behind the meaning of “β-diversity” grows even more… Does it include the matrix? Did you study the relationship between mammal’s presence and mobility on the “seedlings’ β-diversity”?

***Authors: No, we did not sample mammals. We corrected as “Edge contrast is also involved in the inter-patch movement that sustain the persistence of animal seed dispersers and herbivorous mammals within patches [6,40]. Since these animals play a key role in the composition of seedling communities [25,41,42], edge contrast may be affecting β-diversity of seedlings by whether limiting or promoting the presence of seed dispersers, seed predators and herbivorous mammals.” (lines 90-93).***

REVIEWER 2: Lines 100 – 105: There is no such thing as right spatial scale for a species, or for a process. The scale of effect is an approximation that is the result of the interaction between the scales (with emphasis on the plural) of variation of the phenomena that you are observing, with the scale of your observation. In your specific case, there will be a myriad of effects that will simultaneously influence the scales of variation of the phenomena that you are observing and since you are not making a controlled experiment, you don’t have an idea of what are the best scales to try to isolate and observe this or that effect… so it is necessary to apply a method to fine tune your scale of observation to increase the possibility to detect something… this is not the same as to find the “right scale”. To help you clarify the multiple scale hierarchical approach of your work I suggest you begging by reading and citing the two classical works:

- Wu, Jianguo, and John L. David. "A spatially explicit hierarchical approach to modeling complex ecological systems: theory and applications." Ecological modelling 153.1-2 (2002): 7-26.
- Turner M.G., Gardner R.H. (2015) Causes of Landscape Pattern. In: Landscape Ecology in Theory and Practice. Springer, New York, NY. (at least chapter one)

***Authors. Corrected as suggested***

REVIEWER 2: Lines 114 – 115: What about the dispersers’ and herbivorous’ behavior? There are plenty of background information about mammals and birds for example. If they are the bridge that connect landscape pattern to tree seedlings diversity their scale of response may be a good base line, don’t you think?

***Authors: We emphasized the bridge between the dispersers and herbivorous behavior and scales of effect observed in fragmented tropical rainforests, and the possible scale of effect on seedlings diversity in the Methods section (lines 239-244)***

REVIEWER 2: Lines 119 – 124: I am not convinced about the rationale behind your hypothesis one, especially because the α and β diversities were estimated for each patch, what I (and apparently the previews reviewer’s es well) am having a hard time to link with the mechanisms described in the introduction. If you had the samplings made in 16 landscapes composed by multiple patches and the α diversity referred to the richness of each patch and the βdiversity referred to the diversity among patches within each landscape, the logical links established with the functional connectivity of the landscape would be more direct. Since this not the case, it is not clear to me how the spatial heterogeneity within the patch is a function of the landscape context of said patch. In addition, looking to your model selection results as well as the scale selection in the support information you absolutely do not have grounds to assert that there is a difference in scale of response between α and β diversity. Besides, you don’t have a substantial difference in scale extension anyway. I bet that if you look to the correlation among scales for each landscape metric you will see that they are highly correlated, especially the ones that were selected in the model selection process (500, 600 and 800m). Furthermore, because the hierarchical structure of your test is not clear at this point of the text your hypothesis three is also compromised. What specifically are “larger spatial scales” and “local conditions”? How did you access “colonization and extinction dynamics” with your sampling design?

***Authors: We corrected the Introduction to emphasize that we only assessed the effects of landscape composition and configuration on seedlings diversity, because the experimental design does not provide information for assessing landscape connectivity. Therefore, we corrected the lines 122-125 as “Furthermore, we tested the following predictions: (1) α-diversity is influenced by landscape composition and β-diversity by landscape configuration; and (2) there should be larger scales of effect for richness-based metrics than for abundance-based metrics of seedling α- and β-diversity (i.e. dominant < typical < all species)” (lines 123-126). These changes are consistent with the “focal-patch” approach employed in this study. The study design refers the patch as the sample unit, while the landscape is the surrounding environment within a buffer of a radius relevant for a particular biological response (Brennan et al., 2002; Fahrig, 2003). This study approach has effectively linked the patterns and processes occurring within a patch with the composition and configuration of the surrounding landscape (Fahrig, 2013; Watling et al., 2020).***

***In addition, we corrected and highlighted that we did not expect to find differences in the scale of effect between α- and β-diversity. Instead, we emphasized that diversity measures sensitive to abundances are prone to show scales of effect smaller than those diversity measures sensitive to species occurrence. Nevertheless, we are aware that the low substantial differences in scale selection can be associated to a high correlation among scales for each landscape metric. Therefore, we assessed the uncertainty around the selected scale of effect by bootstrapping (lines 277-288). We found a substantial uncertainty in the selected scale of effect among landscape and α-diversity metrics than for β-diversity metrics (Figures S4 and S5), which can explain the lack of differences between scales of effect among α-diversity metrics. We offer a more detailed interpretation regarding the observed scales of effect in the subheading “Scale of landscape effect on diversity metrics” (lines 443-473) in the Discussion section.***

***According to the “habitat amount hypothesis”, the number of species in a plot of fixed size is associated to the total habitat area in the landscape surrounding the plot (Fahrig, 2013).***

-----------------------------------MATERIALS AND METHODS------------------------------------

REVIEWER 2: Lines 129 – 157: In which year the tree seedling sampling was conducted? This information must be provided in the manuscript.

***Authors: We included the year of tree seedling sampling (line 146-147)***

REVIEWER 2: Line 145: It is indispensable that you include a scale bar in Figure 1c, with at least one subdivision showing the exact maximum buffer size.

***Authors: We included the scale bar in Figure 1c***

REVIEWER 2: Lines 165 – 185: The Hill numbers are a very elegant way to decompose diversity, however, it is one approach among many others, such as plain diversity indexes, functional diversity and direct multidimensional community composition comparisons. Therefore, you need to justify why you elected the partition of diversity in α, β and γ combined with Hill numbers approach linking the rational to your theoretical framework in a clearer manner, exposing the causal links and mechanisms that underpin your approach. Despite the noticeable improvements in relation to the manuscript previous versions, from the point of view of the reader this connection is not clear still. I wander what the results for plain patch richness and abundance look like. I suggest you include those.

***Authors: We justified the selection of Hill numbers approach to assess the possible differences in diversity measures at multiple spatial scales in lines 94-112***

REVIEWER 1: Line 206: Consider to write "10 x 10m pixel resolution".

***Authors: Corrected as suggested***

REVIEWER 2: Lines 206 – 212: What is the delay between the satellite imaging and the tree seedlings sampling? The accuracy was accessed in 2013 or when the tree seedlings were sampled?

***Authors: The accuracy was accessed a year before conducting the tree seedling sampling. This delay is due to the availability of SPOT-5 satellite images only for 2013***

REVIEWER 2: Line 215: Density of what? I saw in the table, but your text should be clearer.

***Authors: We corrected as suggested (lines 219-220)***

REVIEWER 2: Lines 216 - 218: The way you are explaining here It is very imprecise. Without looking at the table it is difficult to know if it is the distances of sampled patch to its the neighbors calculated at the patch level or the mean distances among all patches calculated at the landscape level. You should rewrite more precisely.

***Authors: We corrected as suggested (lines 221)***

REVIEWER 2: Line 218: “the percentage of contrasting edges (EC)” is not a traditional description of this metric. I suggest you use the actual name of the index as it is in the table. In addition, the next paragraph should come before the table, since it is fundamental to the comprehension of how the “Edge contrast index” was calculated.

***Authors: We corrected as suggested (lines 222-231)***

REVIEWER 1: Lines 219 - 221. I accept that patch size was not in the models, although I think there are theoretical reasons to think that it could be influential to seedling diversity. In some species, biotic seed dispersal distances are small, and seed dispersal among patches is limited. In those cases, patch size could be relevant regarding seed sources. In my opinion, the justification for disregarding patch size provided in lines 219 - 221 is not convincing. Habitat amount hypothesis is a sound and reasonable hypothesis, still to be tested. Following your line of arguments and the articles cited, isolation would also be weaker than the habitat amount; nevertheless, it was included in the models. However, I understand that we have to choose some explanatory variables to test, and samples are limiting. My suggestion is to improve or remove this justification.

***Authors: We conducted an independent analysis to asses the contribution of patch size and shape to seedling diversity. We found that patch attributes were not relevant on any of the diversity metrics considered in the study. Therefore, we added the following text: “We did neither consider patch size nor patch shape because both variables were highly correlated (r = 0.81), and none of them had significant effects on any of the α- and β-diversity metrics (Table S1)” lines (232-233).”***

REVIEWER 2: Lines 219 – 221: Weaker in your study or in previous ones? This is not a good enough argument to disregard the patch characteristics such as shape and size in your analysis, especially since your definition of landscape is centered in the patch.

***Authors: We corrected as “We then calculated six landscape metrics (Table 1) considered as drivers of seed and seedling community diversity [36,53]. The composition metrics included the percentages of old-growth forest (FC) and secondary forest (SF) covers, whereas the configuration metrics were the number of forest patches per landscape area (patch density; PD), the adjacency among forest patches (aggregation index; AI), the mean distance among all forest patches in the landscape (patch isolation; PI), and the edge contrast index (EC). We calculated the EC using quality values that describe the permeability of different matrix covers for terrestrial mammals. The permeability values were based on the assumption that overall species presence declines along a gradient of habitat loss and relates the percentage of each land-cover type within the landscape matrix to its relative quality [2,6]. We ranked the relative quality of each land-cover type based on the suitability of vegetation structure for mammal feeding, movement and/or habitat on a seven-point scale, including the following: 1 (water bodies, with the lowest suitability); 2 (anthropogenic cover); 3 (cattle pasture); 4 (arboreal crops); 5 (semiaquatic vegetation); 6 (secondary forest); and 7 (old-growth forest, representing the highest suitability). To obtain a more robust and realistic representation of landscape structure effects, we estimated the area-weighted mean of the EC index [74].***

***We did neither consider patch size nor patch shape because both variables were highly correlated (r = 0.81), and none of them had significant effects on any of the α- and β-diversity metrics (Table S1)” lines (217-233).***

REVIEWER 2: Line 223: If I understood correctly, all those indexes were calculated at the class and landscape levels, correct? You should specify this in the text.

***Authors: Corrected as suggested (lines 235-236)***

REVIEWER 2: Lines 237 - 246: What you are calling “large scale” is very modest. The “maximum dispersal distance” is for foraging behavior or migration? What kind of foraging movement these organisms usually do? Are they territorial? Are they gregarious? I understand that the sampled patches are close to each other and that this is a limiting factor to the analysis that you are performing. So, instead of saying that it is the adequate scale for the “species” that you are working with, it is more productive to consider what kind of variation you were able to capture with such sampling design. I am not a specialist in this groups, but seems odd to me that the average movement of birds and primates in such fragmented landscapes would be 4km. In addition, it is necessary distinguish between the variation associated with individual movements from the maintenance of a population of such organisms for example. Both phenomena impact in the measurement that you took despite occurring at different scales. Other complicating factor is that the different kind of dispersers and herbivores most likely have very distinct behaviors and since you did not make a functional analysis your observations are the result of a very complex set of effects, which difficult the association with a scale of analysis of the landscape context. Therefore, it is a bit disingenuous to justify the sampling scales as you did, and I strongly recommend that you change it.

***Authors: Dispersal distance refers to foraging behavior. We corrected as “We followed the recommendation about the landscape radius that best predicts biological responses, which is 0.3–0.5 times the maximum dispersal distance of seed dispersers and herbivore mammals foraging behavior [45,52]. The maximum dispersal distance of arboreal seed dispersers and terrestrial mammals in the study region ranges between 500 and 4000-m [60,80–82].” (lines 239-242).***

REVIEWER 2: Lines 242 – 244: How much overlapping there is in the worst cases? Since there are only 16 patches, it would be a very nice addition a chart 4X4 with the numbered 16 close-ups of the 3000m buffers displaying the 1500m buffers as support information, in addition to a table with the landscape metrics for each patch. This kind of figure contributes a lot for the transparency and can help the reader evaluate the interpretation attributed to each landscape metric. It is also important to show the distance profile of the sampling units (mean distance, distance variance and point aggregation index), the spatial autocorrelation for the landscape metrics as well as the correlation among the metrics themselves. One of the most common mistakes of landscape studies is the incorrect interpretation of the landscape indexes in the context of the study. The landscape metrics are not perfect and not always behavior as originally intended, especially in the more complex ones, such as Shannon’s diversity, shape, edge and most of the configuration ones. Therefore, it is necessary to have a critical view of what they are reflecting in each case.

***Authors: We included the information regarding the distance profile, as well as the overlapping percentage among buffers, the spatial autocorrelation of landscape metrics and the correlation among landscape metrics across the 13 buffer sizes in lines 239-252 and from Table S3 to Table S6 in the Supporting information.***

REVIEWER 2: Lines 281 - 287: You must explain what kind of model you used (lm, glm, glmm,…) with what kind of distribution, present the global model in the text and specify if the models were additive or interactive and what interactions were included if it were the case. If I understood correctly, your N size for each analysis is 16, and your global models have six explanatory variables. It does not make sense to include such big models in the set with sow few samples. It would be best if you reduce the global model size by reduce the number of explanatory variables or constrain the model size combinations that dredge will include in the set. I did not understand why you used two criteria for such simple approach to model selection, since the set is full of redundancy and the parameters were adjusted with very limited sample size. In my opinion you are overcomplicating the model selection and by calculating the “model-averaged parameter estimates” you are introducing more imprecision instead of improving the model. In addition, you must specify how did you calculate “importance and the relative effect” with more detail. Finally, it would be very nice if you include an estimate of pseudo R² or the R² for the adjust between observed X estimated. I did saw that you included R² and R²cv in the model selection table, however if I am not mistaken you did not explain how they were calculated for the model selection.

***Authors: We explained the kind of model and distribution employed, as well as the global models constructed for the model selection procedure, variable importance and relative effect assessments (lines 256-312). This should clarify the misunderstanding about the number of variables employed for each model (the size of the global models was always restricted to the landscape metrics that showed a significant scale of effect, see Table 2).***

------------------------------------------------RESULTS-------------------------------------------------

REVIEWER 2: Lines 299 – 300: This sentence is not sufficiently clear. It should be independent of the figure. Please, rewrite it.

***Authors: Corrected as “We found that α- and β-diversity metrics were significantly higher than the other metrics when species abundances were not considered (Figure S1).”***

REVIEWER 2: Lines 323 – 331: In my opinion this is not a good way to show model selection results. You should at least show the entire set of models with Δ<2 so the reader can check if the null model is not among than. In addition, it is crucial for transparency that you include as support information the tables with the entire rank for each response variable.

***Authors: Corrected as suggested.***

REVIEWER 2: Figure 2c: There is an asterisk absent in AI600.

***Authors: Corrected as suggested***

REVIEWER 2: Lines 796 – 798: The “unconditional standard error (USE)” and its application must be described in the methods section.

***Authors: Corrected as suggested***

---------------------------------------------DISCUSION-----------------------------------------------

REVIEWER 1: Line 432: Substitute "landscape metrics" by "landscape structure". The metrics are just ways to represent the structure.

***Authors: Corrected as suggested***
